# Lead It!: An App to Enable Persons With Dementia to Lead Group Activities for Their Peers

**DOI:** 10.1093/geroni/igaa057.878

**Published:** 2020-12-16

**Authors:** Michael Skrajner, Gregg Gorzelle

**Affiliations:** 1 Hearthstone Alzheimer Care, Wickliffe, Ohio, United States; 2 Hearthstone Alzheimer Care, Hiram, Ohio, United States

## Abstract

LEAD IT! is an app that enables persons with early and middle stage dementia to lead activities for their peers—i.e., other persons with dementia (PWD). An alpha version of the app was tested in a Phase 1 SBIR project. The alpha version included three Montessori-inspired activities. While PWD ostensibly view LEAD IT! as a set of enjoyable activities, it is actually an evidenced-based intervention aimed at reducing responsive behaviors and enabling PWD to fill meaningful social roles. A total of 24 PWD participated in the Phase 1 study: five leaders and 19 players. LEAD IT! Programming was implemented for six weeks, twice per week. LEAD IT! produced higher levels of positive engagement and affect, and lower levels of negative engagement, as compared to standard, baseline activities—i.e., non-digital activities led by staff. More specifically, when compared to baseline programming, players exhibited an 82% increase in Constructive Engagement (P=0.000), 80% increase in Passive Engagement (P=0.000), 60% reduction in Other Engagement (P=0.035), and 171% increase in Pleasure (P=0.000). One limitation of the Phase 1 study is that, at least insofar as the intervention is only implemented twice per week for six weeks, the positive outcomes seem to be limited to the period of time during which PWD are participating in the activity—i.e., changes on global measures, such as quality of life and depression were not detected. Still, the promising results of this study suggest that LEAD IT! is worthy of further development and evaluation in a planned Phase 2 study.

